# Annotations

**Published:** 1887-01-29

**Authors:** 


### Annotations.

"Prudence shall keep thee; discretion shall preserve thee." What a happy world this would be if everybody were moderately wise! Mr. Blundell Maple has offered to the managers of St. Albans Hospital a new site as a free gift. If this offer were accepted, Lord Spencer would contribute £200 to the cost of the new building, and Mr. J. N. Edwards, the town clerk, would "draw the deeds" also free of cost. But—alas that "but" should be so frequently in our vocabulary!—the managers of the Infirmary have looked the "gift horse in the mouth," and have decided that they will not take him, even as a gift. This means that they are throwing away a thousand pounds, or very nearly. What lucky hospital managers those of St. Albans must be, to treat a thousand pounds in this off-hand fashion! Some of the London managers who have new hospital buildings on hand would almost climb to the top of the nearest church steeple, or even of a greased maypole, if they could find £1,000 for their building at the end of their adventure; or if they declined such a feat for themselves, they would at least be willing to let the secretary try his powers, if he were not too old and stout. Joking apart, we cannot but express our surprise at the action of the St. Albans Committee. They have mentioned some objections to the Blundell Maple site; but in London such objections would be laughed at. The proximity of a church and a school ought to be rather welcomed than otherwise, and these seem to be the only avowed obstacles in the way of acceptance. The St. Albans medical men do not appear to have thought them of the slightest importance. There must surely be some other grounds than those named for the strange decision of the Committee. We hope the townspeople will not allow the generous offer which has been made to them to be refused, except for the most clear and convincing reasons.

The Battle of St. Albans.

Birmingham was the birthplace of the Hospital Sunday movement; but, in spite of that honourable distinction, its collections have been slowly diminishing for several years. The fact has excited a creditable feeling of shame in the people of the Midland metropolis, and they have resolved upon better things. Already an influential meeting has been held, and a variety of suggestions offered, for bringing about the desired result. Dr. R. W. Dale, in an effective speech, said that he had, some time ago, read in a professional newspaper, an article with the title: *A new method of extracting gold from reluctant ores.* He was sorry to say the article did not contribute much to the force of his appeal on Hospital Sunday. But he was quite sure that ingenious men might discover many new methods of extracting gold from reluctant ores, and he must say that the best method he had seen yet was an appeal on behalf of the miserable and poor among their fellow-citizens; and when once people began to give, the habit of giving might be confirmed. We trust that the people of Birmingham, and not they only, will be able at least to double the amount of their recent Sunday collections. When all is said, we cannot help feeling that Hospital Sunday makes a poor show compared with what might be done if people would think as much about the poor and sick as they do about their pleasures, and their place in society. The ladies might double their contributions by dispensing annually with a single feather in their bonnets.

Hospital Sunday in Birmingham.

Experimental physiology has a bitter enemy in *The Spectator*. Week after week, and month after month a crusade has been maintained with every variety of literary weapon. Last week an article of a rather hysterical character brought the argument down to date. Whilst we cannot help feeling that *The Spectator* fails to appreciate the scientific instinct, and is much too ready to attribute unworthy motives, we are bound to admit that it is difficult to justify to the public, by positive results, such experiments on living animals as are now made on the Continent, and would be made in this country if the law permitted. After all the labours and researches of physiologists it is still the fact that the hospital ward and the bedside of the private patient are the laboratories which promise the profoundest knowledge of the true art of medicine to the painstaking and conscientious student. The time is past when men despised any kind of knowledge which was merely useful. Knowledge which helps practice is now more valued than any other kind of information. Such knowledge is undoubtedly best gained at the bedside, and in the dead-house. We need not envy the French and Germans their freedom in the practice of vivisection. The patient, diligent, and justifiable use of the best clinical methods will probably keep us fully abreast of the practitioners of the Continent in every department of practical medicine and surgery. If that be so, the knowledge which is merely or mainly curious, and which cannot be gained except at a most terrible cost, may surely be dispensed with.

"The Spectator" and Vivisection.

---

The ills of the feline race do not, as a rule, find medical treatment. If poor puss happens to suffer from asthma, bronchitis, or what not, her recovery is ordinarily left to nature. But puss, when she happens to be the resident of a hospital, is sometimes more fortunately circumstanced. An instance came under our notice the other day. At a hospital at Chelsea, there is a fine handsome-looking tabby which, we were informed, once had the misfortune to get her leg broken. One of the doctors at once set the broken limb, and, to see puss now, you would little fancy that such a severe casualty had ever befallen her.

